# Effects on Puberty of Nutrition-Mediated Endocrine Disruptors Employed in Agriculture

**DOI:** 10.3390/nu13114184

**Published:** 2021-11-22

**Authors:** Anastasia Konstantina Sakali, Alexandra Bargiota, Ioannis G. Fatouros, Athanasios Jamurtas, Djuro Macut, George Mastorakos, Maria Papagianni

**Affiliations:** 1 Department of Endocrinology and Metabolic Diseases, Faculty of Medicine, School of Health Sciences, University of Thessaly, 41110 Larissa, Greece; akonstantina.sakali@gmail.com (A.K.S.); abargio@yahoo.gr (A.B.); 2 Department of Physical Education and Sport Science, University of Thessaly, 42100 Trikala, Greece; fatouros@otenet.gr (I.G.F.); jamurtas@gmail.com (A.J.); 3 Clinic of Endocrinology, Diabetes and Metabolic Diseases, Clinical Center of Serbia, Faculty of Medicine, University of Belgrade, 11000 Belgrade, Serbia; djmacut@gmail.com; 4 Unit of Endocrinology, Diabetes Mellitus and Metabolism, Aretaieion University Hospital, Medical School, National and Kapodistrian University of Athens, 11528 Athens, Greece; mastorakg@gmail.com; 5 Department of Nutrition and Dietetics, University of Thessaly, 42132 Trikala, Greece; 6 Unit of Endocrinology, 3rd Department of Pediatrics, Hippokration Hospital of Thessaloniki, Aristotle University of Thessaloniki, 54642 Thessaloniki, Greece

**Keywords:** nutrition, pesticides, endocrine disruptors, puberty onset, sexual maturation

## Abstract

Pesticide residues are largely found in daily consumed food because of their extensive use in farming and their long half-life, which prolongs their presence in the environment. Many of these pesticides act as endocrine-disrupting chemicals after pre- or postnatal exposure, significantly affecting, among other things, the time of puberty onset, progression, and completion. In humans, precocious or delayed puberty, and early or delayed sexual maturation, may entail several negative long-term health implications. In this review, we summarize the current evidence on the impact of endocrine-disrupting pesticides upon the timing of the landmarks of female and male puberty in both animals (vaginal opening, first estrus, and balanopreputial separation) and humans (thelarche, menarche, gonadarche). Moreover, we explore the possible mechanisms of action of the reviewed endocrine-disrupting pesticides on the human reproductive system. Access to safe, healthy, and nutritious food is fundamental for the maintenance of health and wellbeing. Eliminating the presence of hazardous chemicals in largely consumed food products may increase their nutritional value and be proven beneficial for overall health. Consequently, understanding the effects of human exposure to hazardous endocrine-disrupting pesticides, and legislating against their circulation, are of major importance for the protection of health in vulnerable populations, such as children and adolescents.

## 1. Introduction

Undoubtedly, nutrition should promote human health and a sense of wellbeing and should combat the morbidity and mortality associated with chronic diseases without harming the world ecosystem. However, because the demand for food has grown disproportionately during the last decades because of the increase in the world population, farmers have resorted to an even more intensive application of pesticides to their crops [1]. Multiple pesticides are routinely detected as residues in everyday food products, such as vegetables, fruit, cereals, honey, dairy products, fish and beef meat, and even in human breast milk, sometimes at concentrations exceeding the legally tolerated limits, as established by the Food and Agriculture Organization of the United Nations (FAO) and the World Health Organization (WHO) in their Codex Alimentarius [2,3]. The release on the market, and the legal use, of a pesticide demands approval from an appointed authority, such as the Environmental Protection Agency (EPA) in the US, or the European Commission assisted by the European Food Safety Authority (EFSA) in Europe [4]. Approval demands the meticulous assessment of a large body of studies published in the literature, as well as other studies conducted by the manufacturing company itself, assessing the potential risks and side effects, either acute or delayed, of the pesticide under evaluation with regard to the environment or human and animal health. In Europe, the initial approval lasts for a maximum period of 10 years, and then a renewal is required. Moreover, the EFSA proposes the maximum amount of pesticide residues that are permitted in food, called the maximum residue level (MRL). Each European Union member state is responsible for ensuring the proper use of the approved pesticide inside its territory.

Many among the commonly applied pesticides may exert endocrine-disrupting action in humans and animals after exposure, mainly through the ingestion of contaminated food and, to a lesser extent, through inhalation or dermal absorption [5] (Table 1).

The definition of an endocrine-disrupting chemical (EDC) by the WHO is “an exogenous substance or mixture that alters function(s) of the endocrine system and consequently causes adverse health effects in an intact organism, or its progeny, or (sub) populations” [6]. A significant amount of evidence from clinical, preclinical, and mechanistic studies highlights the role of a wide range of EDCs in affecting the time of puberty onset and the *tempo* of its progression. Puberty is defined as a sequence of events resulting in reproductive capacity. It is characterized by the acceleration of growth velocity and the development of secondary sexual characteristics and genitals. Puberty onset results from the activation of the hypothalamic–pituitary–gonadal (HPG) axis, which remains suppressed during the entire childhood after its initial activation in the fetus and the neonate.

In rodents, the equivalent landmarks of human puberty onset are vaginal opening in females [a strain-dependent event occurring, on average, at postnatal day (PND) 35, and around PND 26 to 30 in rats and mice, respectively], and balanopreputial separation in males (occurring, on average, at PND 43, and around PND 22 to 32 in rats and mice, respectively). On the other hand, in female and male rodents, puberty completion is demarcated by the appearance of the first estrus (i.e., the presence of cornified epithelial vaginal cells) and the presence of mature sperm inside the ductus deferens, respectively [7,8].

The expected age of puberty onset in humans ranges from 8 to 13 years and from 9 to 14 years for girls and boys, respectively. Clinically, it is demarcated by thelarche in girls (the development of breast buds under the areola; Tanner stage 2 in breast development), and by gonadarche in boys (the increase in testicular volume to ≥4 mL, followed by the thinning of the scrotal skin; Tanner stage 2 in external genitalia development) [9,10]. Puberty completion and sexual maturation are considered to occur after the onset of menses (menarche) in girls, and the increase in testicular volume to ≥15 mL in boys. Precocious puberty is defined as the onset of thelarche before the age of 8 years, or the onset of gonadarche before the age of 9 years, in girls or boys, respectively [11]. Precocious puberty is related to a wide range of adverse outcomes, such as an increased risk for breast and ovarian cancer in women, and for testicular and prostate cancer in men, as well as with decreased final height in both sexes. Moreover, it is associated positively with manifestations of psychological disorders, such as depression, as well as with the development of risk-taking behavior, such as alcohol abuse, smoking, drug use, and the early onset of sexual activity which, in turn, increases the risk for sexual abuse [12,13,14,15,16,17]. Early menarche in girls has been associated positively with early natural menopause, an increased risk for uterine fibroids, sleep disorders, diabetes mellitus type 2 (T2DM), obesity, arterial hypertension, angina, osteoarthritis, hiatus hernia, food allergies, and increased all-cause mortality. Early deepening of the voice in boys has been correlated positively with increased risk for arterial hypertension, T2DM, obesity, depression, irritable bowel syndrome, sleep disorders, and poor general health [18,19]. On the other hand, delayed puberty is defined as the absence of thelarche or gonadarche in girls or boys, respectively, occurring at a later age than the population mean by 2 to 2.5 standard deviations (SD) (the age of 13 or 14 years in girls or boys, respectively) [20]. Delayed puberty in humans is associated with lower bone mineral density, increased bone fragility, and an increased incidence of psychological disorders, such as depression [15,21].

## 2. Physiology of Puberty

In humans, the transition to puberty happens through two independent events: adrenarche and gonadarche. Adrenarche, observed only in higher primates (chimpanzees) and in humans, is triggered by an unknown factor preceding gonadarche by approximately two years [22]. The *zona* reticularis of the adrenal cortex matures, resulting in the increased secretion of adrenal androgens. Gonadarche requires an increase in the frequency and amplitude of pulsatile gonadotropin-releasing hormone (GnRH) secretion from the hypothalamic neurons of the preoptic area and the arcuate nucleus, and the subsequent increase of gonadotropins. In boys, follicle-stimulating hormone (FSH) induces the maturation of the gametocytes (spermatogenesis), while luteinizing hormone (LH) stimulates Leydig cells to produce testosterone and androstenedione. In girls, FSH stimulates the proliferation of ovarian granulosa cells, the upregulation of LH receptors, the increase in aromatase activity, as well as the subsequent conversion of testosterone into estradiol, while LH stimulates ovarian theca cells to produce androgens. The trigger for gonadarche remains unknown for either rodents or primates. It is related to various hypothalamic neuropeptides that are involved in the regulation of GnRH pulsatile secretion. These neuropeptides are secreted by kisspeptin-neurokinin B-dynorphin (KNDy) neurons and are either stimulatory (kisspeptin, neurokinin-B), or inhibitory (dynorphin), upon the GnRH neuron [23]. KNDy neurons express sex steroid receptors and are subject to their negative feedback. Apart from the aforementioned neuropeptides, the adipocytokine leptin is capable of modulating the onset and progression of puberty by stimulating kisspeptin production or by suppressing the hypothalamic neuropeptide, Y, a GnRH inhibitor [24].

## 3. Materials and Methods

The role of endocrine-disrupting pesticides upon animal and human puberty physiology, which impacts puberty onset and progression, is reviewed. For the purpose of our review, we searched the PubMed and Google Scholar databases from inception to November 2021, using the algorithm: [pesticides] AND [puberty] AND [menarche OR thelarche OR pubarche OR gonadarche OR “vaginal opening” OR “preputial separation” OR “sexual maturation” OR “testicular enlargement” OR “Tanner stage”]. Additionally, a manual search in the reference lists of the selected review articles was performed. Experimental studies in rodents and primates were included. Exposure was achieved by either the oral or parenteral administration of the examined substance in a variety of dosages, for different periods, and at different stages of development. There were also included observational studies in humans, where exposure to pesticides has been established either directly by detecting the parent compound or its metabolite in biological matrices (serum, urine, cord blood), or indirectly, by confirming occupational or residential exposure with the use of a questionnaire, or by assessing proximity to a given polluted area. Nonoriginal studies, studies in nonmammalian animals, and studies not written in the English language were excluded. Approximately 30% of the retrieved articles claimed no effect of the studied pesticide(s) on puberty onset or progression and, thus, they were not included in the current review.

## 4. Pesticides and Disrupted Puberty Onset or Sexual Maturation

### 4.1. Early Puberty Onset or Accelerated Puberty/Sexual Maturation

#### 4.1.1. Animal Studies 

The published evidence indicates that a substantial number of pesticides widely applied in crops and detected as residues in daily food consumables may significantly accelerate puberty onset and progression (Table 2). In male CD-1 mice, following subcutaneous (sc) injections of the insecticide, cypermethrin, a pyrethroid, from PND 7 to 21 (time at which Leydig cells differentiate and testosterone production increases), accelerated puberty onset, and increased serum gonadotropins and testosterone levels were observed. The exposed animals presented an increased number of Sertoli cells [25]. In summary, postnatal sc exposure to pyrethroids accelerates puberty in male mice, even at a low dose.

The exposure of female rodents to organochlorine insecticides during pre- and either early or later postnatal life has been associated with accelerated puberty. The administration of heptachlor, at a dose of 4.5 mg/kg/day *per os* to female Wistar rats (F0 generation), from gestation day (GD) 12 to lactation day 21, accelerated vaginal opening and increased the incidence of irregular estrous cycles in the F2 generation, which was indirectly exposed via breastfeeding from the F1 generation [26]. The exposure of pregnant Fischer CDF rats to methoxychlor from GD 11 until birth and, thereafter, the parenteral treatment of the female offspring with methoxychlor until PND 7 resulted in accelerated vaginal opening [27]. Female Sprague–Dawley (SD) rats exposed to the greatest dose of methoxychlor (1200 ppm) in their intrauterine and early postnatal life experienced vaginal opening, on average, 2 days earlier compared to controls, while their body weight was significantly lower than that of the controls [28]. Female Long–Evans rats, which received methoxychlor at a variety of doses from their weaning until after their pregnancy, experienced significantly earlier puberty onset and first estrus appearance than their nonexposed peers. Their female offspring, indirectly exposed during intrauterine life and lactation to methoxychlor, experienced earlier vaginal opening as well as a loss of regular estrus cyclicity at the age of 11 months [29]. Similarly, when CD1 female mice were treated from GD 11 to PND 8 with methoxychlor at a very low dosage (20 μg/kg/day), their female offspring experienced earlier vaginal opening and an increase in the number of kisspeptin neurons in the hypothalamus [30]. In female Wistar rats exposed to 10 and 100 mg/kg/day o,p′-dichlorodiphenyltrichloroethane (DDT) sc from PND 6 to 10 (early and short-time exposure), vaginal opening occurred significantly earlier than in controls. First estrus occurred significantly earlier among animals that received 10 mg/kg/day o,p′-DDT sc than in controls [31]. In another study, female SD rats exposed to 1 mg o,p′-DDT sc from PND 2 to 4 presented earlier vaginal opening and earlier first estrus compared to controls [32]. In a similarly designed study, the exposure of female SD rats to 0.1, 0.5, and 1 mg/day o,p′-DDT, from PND 2 to 4, resulted in a dose-dependent acceleration of the time of vaginal opening [33]. Finally, female mouse offspring, exposed from GD 9 to 16 to 15 mg/kg day lindane (γ-HCH) *per os*, experienced, on average, 2 days earlier vaginal opening than nonexposed animals [34]. In summary, methoxychlor accelerates puberty onset in female rats after either gestational or postnatal, or combined gestational and postnatal, oral or sc exposure, even in low doses, while DDT and lindane accelerate puberty onset in female rats after oral or sc postnatal or gestational exposure, respectively. However, heptachlor accelerates puberty onset only in indirectly exposed female rats. With regard to herbicides, animal studies provide evidence for a positive association between the exposure to herbicides and the acceleration of puberty onset in both sexes. Acetochlor, administrated to female Wistar rats at a dose of 7.68 or 15.36 mg/kg/day sc from PND 4 to 7, resulted in approximately 5.22 and 7.32 days, respectively, earlier vaginal opening compared to nonexposed animals [35]. Metolachlor administrated to male Wistar rats from PND 23 to 53 (prepubertal period), at a dose of 5 or 50 mg/kg/day *per os*, resulted in a dose-dependent earlier age of balanopreputial separation [36]. The intraperitoneal administration of 25 mg/kg atrazine from GD 8 to 14 (period of sexual differentiation) in the F0 generation of female SD rats resulted in earlier puberty onset in male and female rats of the F2 and the F3 generation, respectively, compared to nonexposed rats [37]. Lastly, male Wistar rats exposed in utero and during lactation to 50 mg/kg/day glyphosate experienced balanopreputial separation earlier than nonexposed animals [38]. In summary, acetochlor and metolachlor accelerate puberty onset in females and males, respectively, after sc or oral postnatal exposure, while glyphosate accelerates puberty onset in male rats after combined gestational and postnatal exposure. Atrazine accelerates puberty onset in a transgenerational manner in both male and female animals.

#### 4.1.2. Human Studies 

With regard to humans (Table 3), increased urine concentrations of 3-phenoxybenzoic acid (3-PBA), a nonspecific metabolite of 18 different pyrethroid insecticides, among Chinese boys from 9 to 16 years old, were positively associated with accelerated puberty. A 10% increase in 3-PBA concentrations was associated with a 2.4% and 2.9% increase in FSH and LH urine concentrations, respectively [39].

Exposure to organochlorine pesticides is associated with both precocious puberty and uptempo puberty progression among boys and girls. In a study from Belgium, serum hexachlorobenzene (HCB) concentrations among boys 14–15 years old correlated positively with pubic hair development, as well as with testosterone concentrations, while greater serum p-p′-dichlorodiphenyldichloroethylene (p-p′-DDE) concentrations, measured in the same cohort, correlated positively not only with pubic hair, but also with gonadal development [40]. In another study, serum concentrations of DDE and polychlorinated biphenyls, measured from 1973 to 1991, in women residing near Lake Michigan (USA), which was highly polluted with industrial waste products, were positively associated with the earlier menarche of their in-utero-exposed daughters [41]. Accordingly, among 466 Chinese women (20–36 years old) who reported early menarche, those with serum DDT concentrations at the highest quartile had experienced menarche, on average, 1.11 years earlier than those with serum DDT concentrations at the lowest quartile [42]. Moreover, in a cohort of boys 14 to 15 years old, a double increase in the serum concentration of HCB was related to a 3-fold possibility of achieving a stage 3 genital development in the Tanner scale of pubertal maturation, and with a 4-fold possibility of achieving a stage 3 pubic hair development, while a double increase in the serum concentration of p-p′-DDE was related to a 1.5-fold possibility of achieving a stage 3 of pubic hair or genital development. In an adjusted model of the simultaneous exposure to other pollutants, statistical significance remained only for the association between serum HCB concentrations and pubic hair development, as well as for the association between p-p′-DDE and genital development [43]. In a study examining idiopathic precocious puberty, affected children had significantly increased odds of presenting detectable serum p-p′-DDE concentrations compared to nonaffected children [44]. In Belgium, among girls with idiopathic precocious puberty, those having migrated from Asia, Africa, South America, and Western Europe had approximately 10 times greater serum concentrations of p,p′-DDE than those belonging to the indigenous population [45]. In a study from Denmark, daughters born to female greenhouse workers, exposed occupationally to a mixture of pesticides during their first trimester of pregnancy, experienced thelarche significantly earlier (on average, at 8.9 years of age) than daughters born to nonexposed female workers (on average, at 10.4 years of age) [46].

Herbicides have been positively associated with earlier sexual maturation in girls. Concentrations of diaminochlorotriazine (DACT) in the urines of mothers, collected during their respective pregnancies, were greater in those of girls who presented menarche before 11.5 years of age, compared to mothers of girls who presented menarche later than 11.5 years [47].

### 4.2. Late Puberty Onset or Delay in Puberty Progression/Sexual Maturation

#### 4.2.1. Animal Studies 

The oral administration of esfenvalerate, a pyrethroid insecticide, in female prepubertal SD rats (at PND 22 or 29) at different doses resulted in delayed vaginal opening (1 or 2 days, respectively) in the animals exposed to 1 or 5 mg/kg/day, compared to nonexposed animals, as well as in decreased serum estradiol concentrations (Table 2). All exposed animals exhibited a suppression of the normal evening rise in the serum LH concentration; however, neither the baseline FSH and LH concentrations, nor their pituitary responses to LHRH administration, were affected [48]. In addition, male rats, whose mothers were exposed to 1 or 25 mg/kg/day cypermethrin, a pyrethroid, during pregnancy and lactation experienced a significant delay in testicular descent and balanopreputial separation (on PNDs 38–42 and PNDs 39–45, with respect to the administrated doses) as compared to nonexposed rats (PNDs 35–37). The exposed animals with delayed puberty onset demonstrated reduced prepubertal body weight. Moreover, the animals exposed to the greatest dose of cypermethrin had decreased sperm mobility and numbers, decreased serum testosterone, and increased serum estradiol concentrations. Additionally, researchers found the disrupted expression of steroid hormone receptors in the testes of exposed animals [49]. Similarly, female rats, whose mothers were treated with 25 mg/kg cypermethrin during pregnancy and lactation, experienced a significant delay in vaginal opening compared to nonexposed rats [50]. In summary, pyrethroids delay puberty onset in male and female animals after per os postnatal, or combined gestational and postnatal, exposure, even at low dosages. Organochlorine insecticides, in several studies, significantly delayed puberty onset when administrated to male animals. Few studies reported a comparable outcome when organochlorines were administrated to female animals. For instance, a significant delay in the time of vaginal opening was observed among female SD rats exposed at a dose of 30 μg/kg/day to the organochlorine pesticide, heptachlor, during intrauterine life, lactation, and postweaning, until PND 42 [54]. In addition, when heptachlor was administrated to female rats (F0 generation) during the second half of their pregnancy and breastfeeding, their offspring (F1 generation) experienced a delay in the time of vaginal opening by 2 days, on average, compared to nonexposed animals [24]. Male Holtzman rats, exposed in utero from GDs 14 to 18 (sexual differentiation time) to the greatest p-p′-DDT dose (200 mg/kg/day), experienced balanopreputial separation significantly later (PND 44.22 ± 0.62), compared to nonexposed controls (PND 42.48 ± 0.29) [51]. Male rats that received DDE at a high dosage (100 mg/kg/day), from weaning until PNDs 55–56, experienced balanopreputial separation, on average, 2.1 days later than the control animals. Nevertheless, no difference in the time of puberty onset was observed among animals exposed later in life (from PNDs 35–36 until 55–56) and controls [52]. Male SD rats exposed to the greatest dose of methoxychlor (1200 ppm) in their intrauterine and early postnatal life experienced balanopreputial separation, on average, 2 days later than the controls (PND 43.3 ± 1.6 vs 41.3 ± 1), while, at the same time, they had significantly lower testicular weights on the day of weaning [26]. In another study, methoxychlor was administrated per os to male Long–Evans rats from the day of weaning until PND 80. Among these rats, those exposed to the greatest dosages of methoxychlor (100 and 200 mg/kg/day) experienced a significant delay in the time of balanopreputial separation (PNDs 43.8 and 53 regarding the respective dosages, compared to PND 40.4 for the nonexposed animals) [27]. Similarly, when Long–Evans male rats were given 100 mg/kg/day methoxychlor per os from PND 21 to 57, the time at balanopreputial separation was delayed by 5 days compared to the controls [53]. Delay in puberty onset in a dose-dependent manner was also observed among male SD rats indirectly exposed to methoxychlor at conception time, in utero and during lactation [55]. Moreover, when CD1 female mice were treated from GD 11 to PND 8 with methoxychlor at a very low dosage (20 μg/kg/day), their male offspring experienced balanopreputial separation later than nonexposed animals [30]. In summary, exposure to organochlorine insecticides during intrauterine and postnatal life delayed puberty onset among male animals.

Evidence of a positive association between exposure to herbicides and the delay in pubertal timing among both sexes of the tested animals emerges from numerous studies. In one of them, female offspring SD rats treated during gestation (GD 14 to 21) with the herbicide, atrazine, at a high daily dose (100 mg/kg/day), exhibited a delay of 1.6 days in vaginal opening [56]. Likewise, female Long–Evans rats exposed in utero to the same atrazine dosage for 7 days (from GD 13 to 19) experienced a 2-day delay in vaginal opening, while their serum total testosterone concentration at the onset of puberty, and throughout adult life, was two times greater than in nonexposed animals [57]. In another study, where male Wistar rats were treated with atrazine from PND 23 to 53–54 at different dosages (12.5, 50, 100, 150, and 200 mg/kg/day), balanopreputial separation happened later by 1.6 to 3 days in all exposed groups compared to nonexposed animals. The group of animals treated with the greatest atrazine dosage (200 mg/kg/day) had decreased intratesticular testosterone concentrations and increased estradiol and estrone serum concentrations compared to nonexposed animals [61]. Correspondingly, when female SD rats were exposed during intrauterine life and thereafter to 25 and 50 mg/kg/day atrazine, they experienced a delay in vaginal opening by 1 or 2 days, respectively. Delays in vaginal openings were also observed among rats treated with 50 mg/kg/day atrazine after weaning [68]. When atrazine was administrated to female Wistar and SD rats from PND 21 continuously for 25 days, a 3-day delay of the onset of puberty was observed among the Wistar rats exposed to the greatest atrazine dosage (100 mg/kg/day), and among SD rats exposed to the median and the greatest atrazine dosages (30 and 100 mg/kg/day) [63]. Delay in balanopreputial separation was observed among male Wistar rats treated from PND 23 to 53 with the atrazine metabolite, DACT, at a dosage equivalent to ≥12.5 mg/kg/day atrazine, as well as with the atrazine metabolites, deethylatrazine (DEA) or deisopropylatrazine (DIA), at a dosage equivalent to 25, 100, or 200 mg/kg/day atrazine. The animals exposed to the two greatest DACT dosages had increased estrone serum concentrations on PND 53, while those exposed to the two greatest DIA dosages had decreased serum testosterone concentrations [62]. Female Wistar rats treated with atrazine from PND 22 to 41, at dosages ≥ 50 mg/kg/day, experienced a delay in the time of vaginal opening. Delays in vaginal openings were also observed among animals treated with 106.7 or 213 mg/kg/day propazine, or ≥33.8 mg/kg/day DACT [64,65]. Male SD rats exposed in utero, from GD 14 until birth, to 50, 75, or 100 mg/kg/day atrazine, experienced balanopreputial separation later than nonexposed male rats. The rats exposed to 75 and 100 mg/kg/day had, on PND 60, decreased serum testosterone concentrations, while the animals exposed to 50, 75, and 100 mg/kg/day had, on PND 60, decreased intratesticular testosterone concentrations [60]. Furthermore, Long–Evans rats exposed in utero, from GD 15 to 19, to either 100 mg/kg/day atrazine, or to a 0.87 mg/kg/day or 8.73 mg/kg/day mixture of atrazine metabolites (HA, DACT, DIA, DEA) experienced a significant delay in puberty onset [58,59]. Among female Wistar rats treated with the herbicide, simazine, for 21 days, starting from the day of weaning, those who received simazine at 25 or 100 mg/kg/day experienced vaginal opening significantly later than the nonexposed animals. Among animals treated for 41 days, those who received simazine at a dosage ≥ 25 mg/kg/day experienced vaginal opening significantly later than the nonexposed animals. The animals that received the greatest simazine dosages (100–200 mg/kg/day), either for 21 or for 41 days, experienced significant delays in the time of first estrus [66]. A glyphosate-based herbicide named Roundup, when administrated to SD female rats (F0 generation) from GD 6 until the end of breastfeeding, and to their female offspring (F1 generation) from weaning until adult life, resulted in the delayed appearance of the first estrus in the F1 generation, and to increased serum testosterone concentrations at adulthood [69]. Moreover, male Wistar rats treated with 50 and 250 mg/kg/day glyphosate, from PND 23 to 53, experienced a delay in balanopreputial separation in a dose-dependent manner [67]. In summary, atrazine delayed puberty onset in male animals after gestational or oral postnatal exposure at dosages above the no-observed-adverse-effect level (NOAEL), and glyphosate delayed puberty onset in male animals after per os postnatal exposure, even at the equivalent to the NOAEL dosage. Moreover, glyphosate at a high dosage delayed the sexual maturation of female animals exposed during gestation and breastfeeding.

Fungicides were found, in several studies, to significantly delay the onset of puberty among male animals. In one study, prochloraz was administrated per os to female Wistar rats from GD 6 until the end of lactation, and to their male and female offspring from weaning until PND 83, at the dosages 0.01, 5, and 30 mg/kg/day. Male offspring exposed to the greatest dosage experienced a delay in balanopreputial separation by 1 day. Additionally, male offspring exposed to the medium and greatest dosages exhibited temporary nipple retention on PND 12, compared to nonexposed rats that had already lost their nipples [72]. In a different study, in which male SD rats were treated from PND 23 to 42 with 31.3, 63.5, or 125 mg/kg/day prochloraz, those exposed to the greatest dosage experienced a significant delay in the day of onset and the completion of balanopreputial separation. With all dosages, exposed animals had, at the end of treatment, significantly decreased serum testosterone concentrations [70]. Male Wistar rats exposed to a mixture of vinclozolin, flutamide, and prochloraz at a low dosage (20, 0.25, or 30 mg/kg/day, respectively), in utero from GD 6 to 20, and, additionally, from weaning until puberty, experienced a significant delay in balanopreputial separation by approximately 10 days [71]. In summary, fungicides delayed puberty onset among male animals after postnatal exposure at high dosages orally, or after combined intrauterine and postnatal exposure.

#### 4.2.2. Human Studies 

In girls, pyrethroids are associated with delayed puberty, as shown in a cohort of Chinese girls aged 9–15 years old, by the positive association between both delayed age at menarche and slower puberty progression, and the increased 3-PBA concentrations in their urine [73] (Table 3).

Organochlorine pesticides, in human studies, have been found to significantly delay puberty onset and progression among boys, similar to the effect observed in animals. Few studies report a similar outcome among girls. In a study conducted in the Russian industrial city of Chapayevsk, where factories handle organochlorine chemicals, serum HCB concentrations in prepubertal boys (aged 8–9 years old), measured at the greatest quartile, were associated positively with a significant delay (by approximately 5 months) in the time of sexual maturation (defined according to a testicular volume ≥ 20 mL) [74].

In a Belgian study, serum HCB and p-p′-DDE concentrations among girls 14–15 years old were associated positively with delayed age at thelarche and menarche [40]. In a study from northern Kazakhstan, researchers examined puberty progression among 517 girls living in two distinct areas: The first area was characterized by dense cotton cultivations, where 20 different organochlorine pesticides were applied intensively until after 2007, when the Stockholm convention banned their use. The second area was characterized by the predominance of livestock farming. Serum organochlorine concentrations were significantly increased among girls living in the cotton farming area. Moreover, these girls exhibited a delayed puberty progression rate, as well as delayed age at menarche compared to the girls living in the livestock farming area. Additionally, FSH, LH, estradiol and insulin-like growth factor-1 (IGF-1) serum concentrations, height, and ovarian volume were all decreased among the exposed girls in all age categories, compared with the nonexposed girls [75]. Similarly, in a study from the USA, serum organochlorine pesticides, measured in the greatest quartile among girls enrolled at age 6–8 years old, were associated positively with delayed menarche [78]. Furthermore, the intrauterine exposure of male fetuses to p-p′-DDE was associated negatively with serum LH and testosterone concentrations at 14 years of age, as well as with puberty progression [76]. Researchers examined the impact of endosulfan exposure among 117 boys, 10–19 years old, who lived in an area where endosulfan was sprayed intensively over a period of 20 years, as compared to a group of 90 boys of the same age living in a different area without endosulfan air contamination. Endosulfan was detected in the serum of 78% of the boys living in the exposed area, in significantly greater mean concentrations (7.47 ± 1.19 ppb), compared to 29% of the boys living in the nonexposed area (1.37 ± 0.4 ppb endosulfan). The boys who lived in the contaminated area had lower Tanner pubic hair and genital development scores, lower serum testosterone, and greater serum LH concentrations when compared to the boys who lived in the noncontaminated area [77]. Regarding organophosphate pesticides, in a Belgian study, urine methyl or ethyl metabolite concentrations were associated positively with delayed genital development among boys 14–15 years old, or with delayed breast development among girls 14–15 years old, respectively [40].

In an already aforementioned Danish study, sons born to female greenhouse workers occupationally exposed to a mixture of pesticides during their first trimester of pregnancy had smaller penile lengths and testicular volumes, assessed by testicular ultrasounds at 3 months of age and at prepubertal age, than sons of the same age born to nonexposed female workers. Furthermore, boys exposed in utero did not have significantly increased serum dehydroepiandrosterone sulfate (DHEA-S) and Δ4-Androstenedione concentrations at prepubertal age, compared to nonexposed boys, while the serum FSH, LH, prolactin (PRL), and testosterone concentrations were comparable between the examined groups [79].

## 5. Discussion

### Mechanisms of Endocrine-Disrupting Action of Pesticides in Puberty Physiology

To summarize the observed actions of EDCs in animals and humans, exposure to pyrethroid insecticides (esfenvalerate) was found to delay puberty onset among female rodents exposed prepubertally, as well as to delay puberty progression and menarche among exposed girls. On the other hand, interestingly, exposure to pyrethroid insecticides (cypermethrin) was found to accelerate puberty onset among male mice exposed postnatally, and to delay puberty onset among male rats exposed either pre- or postnatally. The effects of pyrethroids were evident after postnatal, or combined gestational and postnatal, exposure, even at levels below the NOAEL for reproductive toxicity. Among boys, exposure to pyrethroids was associated with an acceleration of puberty progression. Organochlorine insecticides (DDT/DDE, methoxychlor, lindane) were found mainly to accelerate and delay puberty onset among female and male rodents, respectively, exposed to them either pre- or postnatally. With regard to methoxychlor, its effect on male or female puberty was evident at exposure levels well below the NOAEL for reproductive toxicity. Interestingly, female rats exposed prenatally to heptachlor experienced a delay in puberty onset, although puberty onset among their female offspring was accelerated. In humans, organochlorine pesticides were associated with earlier puberty onset and with either delayed or accelerated pubertal progression among both girls and boys. Among herbicides, atrazine was found to delay puberty onset among both female and male rats exposed to this herbicide, either pre- or postnatally. Simazine and acetochlor delayed and accelerated puberty onset and progression, respectively, in female rats exposed to these herbicides postnatally. Puberty onset among male rats exposed to herbicides was either delayed (atrazine, glyphosate) or accelerated (metolachlor, glyphosate). The effects were observed at exposure levels equivalent to, or above, the NOAEL for reproductive toxicity with regard to glyphosate and atrazine, respectively. In women, prenatal exposure to herbicides was associated positively with earlier menarche. Fungicides were found to delay puberty onset among male rats after postnatal, or combined gestational and postnatal, exposure, at levels well above the NOAEL for antiandrogenic effects, but no impact was observed on the puberty onset of female rats. Finally, human exposure to organophosphates resulted in the delayed sexual maturation of both boys and girls. Discrepancies among animal studies, in which it is difficult to identify a pattern of experimental conditions associated to the effects of pesticides on puberty, originate mainly from the different study designs (time, duration, route of exposure, dosages). On the other hand, discrepancies among human studies result from the possible synergistic effect of other endocrine-disrupting pesticides, compounds, or environmental factors, as well as from the genetic variability of the populations studied. Many in vitro and in vivo animal studies have highlighted the mechanisms of the action of EDCs on the reproductive system by indicating their toxic effects, such as the alteration of the anogenital distance at birth (a biomarker of intrauterine androgen exposure) [80], disruption at the time of puberty onset and progression, reduced fertility during reproductive life [81], negative pregnancy outcomes [82], and an increased incidence of reproductive tract disorders and malignancies [83]. Such mechanisms include: (a) The binding to sex steroid hormone receptors (androgen receptor AR and estrogen receptor ER), resulting in an agonistic or antagonistic (to the corresponding hormone) action, depending on whether they activate or inactivate the hormonal response; (b) The inhibition or stimulation of the enzymic action of key steroidogenic enzymes, such as aromatase or 5α-reductase; (c) The binding to the aryl hydrocarbon receptor (AhR), resulting in the disrupted regulation of genes involved in reproductive processes, such as estradiol production; (d) Epigenetic alterations, such as histone modifications, DNA methylation, or the production of noncoding RNAs, which change gene expression in an heritable manner without altering the DNA sequence; (e) Disruption of the hypothalamic or pituitary functions, resulting in changes in the GnRH and gonadotropin pulsatile secretions; (f) An imbalance between antioxidants and pro-oxidant molecules, resulting in the increased production of pro-oxidant molecules, thus negatively affecting puberty progression, the quality of gametocytes, and pregnancy outcome; (g) Disruption of the cell cycle progression, resulting in uncontrolled cell proliferation or apoptosis [84,85].

Each endocrine-disrupting pesticide may employ one, or multiple, mechanisms of action. For instance, among pyrethroids, cypermethrin impairs the maturation of the HPG axis by interacting with the sodium and calcium channels in hypothalamic, pituitary, and testicular cells. In addition, it disrupts the expression of various genes coding for enzymes, such as steroidogenic acute regulatory protein (StAR) and the cholesterol side-chain cleavage enzyme (CYP11A1) inside male gonadal cells, as well as the genes regulating the production of the FSH and LH subunits [23]. Similarly, fenvalerate disrupts the activity of the steroidogenic enzymes, CYP11A1 and 17 beta-hydroxysteroid dehydrogenase (17β-HSD), inside female gonadal cells [86]. On the other hand, esfenvalerate is capable of disrupting gonadotropin synthesis, acting at the level of the central nervous system [48].

Among organochlorine pesticides, methoxychlor acts as an agonist for the estrogen receptor subtypes, ERα and Erβ, and as an antagonist for AR. In an in vitro study, the serum of the exposed-to-methoxychlor female rhesus monkeys exhibited increased estrogenic activity and remained estrogenic even after the precipitation of the endogenous estrogen with the appropriate antibody [87]. Methoxychlor suppresses the expression of aromatase, 17α-hydroxylase/17,20-lyase (CYP17a1), and the CYP11A1 and 17β-HSD enzymes, thus inhibiting steroidogenesis. Methoxychlor acts epigenetically via DNA methylation in the ERβ gene promoter region [85]. In addition, methoxychlor either down- or upregulates the expression of several genes in the hypothalamic arcuate nucleus, which contribute to the regulation of energy homeostasis and female reproductive capacity (adiponectin receptor 1, muscarinic acetylcholine receptor 3, 5-hydroxytryptamine receptor, and the IGF-1 genes) [25]. Endosulfan binds to ER, thus increasing estrogenic activity. In in vivo and in vitro experiments, endosulfan causes ovarian regression as well as the downregulation of genes associated with the male reproductive system, respectively. [85]. In addition, the organochlorine pesticide, lindane, acts as an estrogen agonist via binding to ERβ. Similarly, DDT acts as an ER agonist, while its metabolite, DDE, is an AR antagonist [53]. Moreover, it decreases the interval between the GnRH pulses, as seen in hypothalamic transplants cultured with DDT [31]. Furthermore, HCB is a ligand to AhR, acting by inhibiting androgen production and AR binding [74].

Among herbicides, atrazine stimulates aromatase activity because male animals exposed to this herbicide had decreased testicular testosterone, but increased serum estradiol and estrone concentrations [61]. Additionally, atrazine causes epigenetic alterations, as suggested by the presence of the DNA methylation regions found in the spermatozoa of three generations of animals (directly exposed animals, as well as the offspring of the following two generations) [37]. Furthermore, atrazine suppresses LH and PRL secretion in ovariectomized female animals, an effect possibly mediated by the observed alterations in the secretion of the neurotransmitters, norepinephrine and dopamine [88]. On the other hand, females exposed to atrazine prenatally had increased serum androgen concentrations at puberty and adult life [58]. The intrauterine exposure of female animals to the herbicide, glyphosate, led to increased serum androgen concentrations and androgenization [69]. Glyphosate suppressed aromatase activity in placental and embryonic human cells [89,90]. Increased pituitary and serum LH concentrations, as well as increased pituitary LH mRNA expression, among male rats exposed prenatally to glyphosate have been reported [38]. The herbicide, acetochlor, increased the number of ERs in the uteruses of ovariectomized animals [35]. Finally, the herbicide, metolachlor, stimulated aromatase activity in the cultures of human cells [91].

Among fungicides, prochloraz acts as both an AR antagonist and as an aromatase and 17α-hydroxylase inhibitor, leading to the suppression of steroidogenesis and testosterone production [70].

Among organophosphate pesticides, one of the suggested mechanisms of the reproductive toxicity of chlorpyrifos is the oxidative damage exerted in the anterior pituitary and Leydig cells, resulting in a decreased production of gonadotropins and testosterone [92].

Overall, a significant amount of evidence from animal (Table 2) and human (Table 3) studies suggests the disrupting effect of pesticides, widely applied in farming nowadays, on the reproductive system. These pesticides are commonly detected as residues in daily consumable foods, such as milk, honey, grain products and, mainly, fruit and vegetables. Since it is recommended that fruit and vegetables be consumed in large amounts daily because of their established benefits for cardiovascular health and other metabolic parameters, the danger of being continuously exposed to hazardous chemicals lurks. Thus, more studies on humans must be conducted to establish solid connections between exposure to EDCs via nutrition and the reproductive health effects [93].

The availability of food free from hazardous chemicals promotes health, a sense of wellbeing, and prosperity. According to a meta-analysis, food contaminated with significantly lower pesticide levels carries higher antioxidant properties and is nutritionally beneficial for human health [94]. Another systematic review highlights the positive association between organic food consumption and a reduced incidence in a number of negative health outcomes [95]. Nowadays, new technologies have emerged with regard to the production of safer pesticides, such as genetically engineered plants with insecticide properties, and pesticides encapsulated or fixed in nanomaterials (nanopesticides) that are designed to enhance safety and efficacy by preventing their premature degradation and ensuring their controlled and targeted release. Moreover, farmers are starting to use alternatives to the use of pesticide solutions, such as biopesticides (e.g., bacteria, fungi with insecticidal properties, or pesticides having natural substances as active ingredients). Access to safe and healthy food is an established human right. Therefore, the application of pesticides should take into consideration not only the need for greater crop yields, but also the long-term environmental and human health consequences.

## Figures and Tables

**Table 1:** Categories of endocrine-disrupting pesticides reviewed.

Pesticide Category	Agrochemical Substance	Metabolites
**Insecticides**		
*Pyrethroids*	esfenvalerate, cypermethrin	3-PBA
*Organochlorines* (*POPs*)	heptachlor, DDT (banned in EU, USA), methoxychlor (banned in USA), endosulfan (banned in EU, USA), lindane, dieldrin, endrin	DDE
*Organophosphates* (*potential POPs*)	chlorpyrifos (banned in EU, USA)	DMP, DMTP, DMDTP, DEP, DETP, DEDTP
**Herbicides**	atrazine (banned in EU), propazine, simazine (banned in EU), acetochlor (banned in EU), metolachlor, glyphosate	HA, DACT, DIA, DEA
**Fungicides**	prochloraz, vinclozolin (reprotoxic, banned in EU), HCB (banned in EU, USA)	

3-PBA: 3-phenoxybenzoic acid; POP: persistent organic pollutant; DDT: dichlorodiphenyltrichloroethane; EU: European Union; USA: United States of America; DDE: dichlorodiphenyldichloroethylene; DMP: dimethylphosphate; DMTP: dimethylthiophosphate; DMDTP: dimethyldithiophosphate; DEP: diethylphosphate; DETP: diethylthiophosphate; DEDTP: diethyldithiophosphate; HA: hydroxyatrazine; DACT: diaminochlorotriazine; DIA: deisopropylatrazine; DEA: deethylatrazine; HCB: hexachlorobenzene. Information on banned pesticides available at: https://pan-international.org/pan-international-consolidated-list-of-banned-pesticides (accessed on 16 November 2021).

**Table 2:** Animal studies showing effects on puberty.

Publications	Agrochemical Substance	Animal	Period of Exposure	Dosage	Impact on Puberty Landmarks	NOAEL for Reproductive Toxicity
	**Insecticides**					
	*Pyrethroids*					
			*Postnatal*			
Pine et al., 2008 [48]	Esfenvalerate	Female SD rats	PND 22–VO	0.5, 1 or 5 mg/kg/day per os	VO delay at 1 and 5 mg/kg/day	2 mg/kg/day
Ye et al., 2017 [25]	Cypermethrin	Male CD-1 mice	PND 7–PND 21	0.5, 5 or 50μg/kg/day sc	Acceleration of PPS at all dosages	5 mg/kg/day
			*Gestational and Postnatal*			
Singh et al., 2017 [49]	Cypermethrin	Holtzman rats	GD 6–LCD 21	1, 10 or 25 mg/kg/day per os	Delay of PPS at 1 and 25 mg/kg/day	5 mg/kg/day
Singh et al., 2020 [50]	Cypermethrin	Holtzman rats	GD 6-LCD 21	1, 10 or 25 mg/kg/day per os	Delay of VO at 25 mg/kg/day	5 mg/kg/day
	*Organochlorines*					
			*Gestational*			
Loeffler and Peterson 1999 [51]	DDT	Holtzman rats	GD 14–GD 18	1, 10, 50, 100, or 200 mg/kg/day per os	PPS delay at 200 mg/kg/day	n/a
Maranghi et al., 2007 [34]	Lindane	CD1 mice	GD 6–GD 16	15 mg/kg/day per os	VO acceleration	n/a
			*Postnatal*			
Rasier et al., 2007 [31]	ο,p′-DDT	Female Wistar rats	PND 6–PND 10	10 or 100 mg/kg/day sc	VO acceleration at all dosages, acceleration of first estrus appearance at 10 mg/kg	n/a
Heinrichs et al., 1971 [32]	ο,p′-DDT	Female SD rats	PND 2–PND 4	1 mg/day sc	Acceleration of VO and of first estrus appearance	n/a
Gellert et al., 1974 [33]	ο,p′-DDT	Female SD rats	PND 2–PND 4	0.001, 0.01, 0.1, 0.5, or 1 mg/day sc	Dose-dependent VO acceleration at ≥0.1 mg/day	n/a
Ashby and Lefevre 2000 [52]	DDE	Male Alderley Park rats	PND 22–55 or PND 36–55	100 mg/kg/day per os	PPS delay in the PND 22–55 subgroup	n/a
Kelce et al., 1995 [53]	Methoxychlor	Male Long–Evans rats	PND 21–PND 57	100 mg/kg/day per os	PPS delay	n/a
			*Gestational and Postnatal*			
Martinez-Ibarra et al., 2016 [26]	Heptachlor	Wistar rats	F0 generation: GD 12–LCD 21	4.5 mg/kg/day per os	F1 generation: VO delay F2 generation: VO acceleration	n/a
Smialowicz et al., 2001 [54]	Heptachlor	SD rats	GD 12–LCD 7 PND 8–PND 42	0, 30, 300, or 3000 μg/kg/day per os	VO delay at 30 μg/kg/day	n/a
Masutomi et al., 2003 [28]	Methoxychlor	SD rats	GD 15–LCD 10	24, 240, or 1200 ppm/day per os	VO acceleration and PPS delay at 1200 ppm	n/a
Roepke et al., 2016 [27]	Methoxychlor	Fischer CDF rats	Mothers: GD 11–PND 0 Female offspring: PND 0–PND 7	75 mg/kg/day intraperitoneally to the pregnant dams, sc to the neonates	VO acceleration	n/a
Martini et al., 2020 [30]	Methoxychlor	CD1 mice	GD 11–LCD 8	20 μg/kg/day per os	Acceleration of VO in female offspring, delay of PPS in male offspring	5 mg/kg/day
			*Postnatal and Adult*			
Gray et al., 1989 [29]	Methoxychlor	Male and female Long–Evans rats	PND 21–PND 80 (males) PND 21–LCD 15 (females)	25, 50, 100, or 200 mg/kg/day per os	F0 generation: Acceleration of VO and of first estrus appearance at all dosages; PPS delay at 100 or 200 mg/kg/day F1 generation: VO acceleration at all dosages.	n/a
Aoyama et al., 2012 [55]	Methoxychlor	Female and male SD rats	From postnatal week 5 and for 18 weeks	10, 500, or 1500 ppm per os	PPS delay at 500 and 1500 ppm.	10 ppm
	**Herbicides**					
			*Gestational*			
Davis et al., 2011 [56]	Atrazine	SD rats	GD 14–GD 21	1, 5, 20 or 100 mg/kg/day per os	VO delay at 100 mg/kg/day	n/a
Rayner et al., 2005 [57]	Atrazine	Long–Evans rats	GD 13–15; GD 15–17; GD 17–19; GD 13–19	100 mg/kg/day per os	VO delay in the GD13–19-exposed group	n/a
Rayner et al., 2007 [58]	Atrazine	Long–Evans rats	GD 15–GD 19	100 mg/kg per os	PPS delay among offspring exposed in utero and throughout lactation	n/a
Stanko et al., 2010 [59]	Mixture of atrazine and its metabolites (HA, DACT, DIA, DEA)	Long–Evans rats	GD 15–GD 19	0.09, 0.87, or 8.73 mg/kg/day of the mixture or 100 mg/kg/day atrazine per os	PPS delay among offspring exposed to 0.87 or 8.73 mg/kg/day of the mixture or 100 mg/kg/day atrazine	6.25 mg/kg/day for DACT
Rosenberg et al., 2008 [60]	Atrazine	SD rats	GD 14–PND 0	1, 10, 50, 75, or 100 mg/kg/day per os	PPS delay at 50, 75, or 100 mg/kg	n/a
			*Postnatal*			
Stoker et al., 2000 [61]	Atrazine	Male Wistar rats	PND 23–PND 53	12.5, 25, 50, 100, 150, or 200 mg/kg/day per os	PPS delay at 12.5, 50, 100, 150, or 200 mg/kg/day	6.25 mg/kg/day
Stoker et al., 2002 [62]	Atrazine metabolites (DEA, DIA, DACT)	Male Wistar rats	PND 23–PND 53	6.25, 12.5, 25, 50, 100, or 200 mg/kg/day per os in molar equivalent of atrazine	PPS delay in subgroups which received DEA or DIA (at 25, 100, and 200 mg/kg) or DACT (at ≥12.5 mg/kg)	6.25 mg/kg/day for atrazine and DACT, 12.5 mg/kg/day for DEA and DIA
Ashby et al., 2002 [63]	Atrazine	Female Wistar and SD rats	PND 21–PND 45	10, 30, or 100 mg/kg/day per os	Wistar rats: VO delay at 100 mg/kg/day SD rats: VO delay at 30 or 100 mg/kg/day	25 mg/kg/day
Laws et al., 2000 [64]	Atrazine	Female Wistar rats	PND 22–PND 41	12.5, 25, 50, 100, or 200 mg/kg per os	VO delay at 50, 100 or 200 mg/kg	25 mg/kg/day
Laws et al., 2003 [65]	HA or DACT (Atrazine metabolites) or Propazine	Female Wistar rats	PND 22-PND 41	22.8, 45.7, 91.5, or 183 mg/kg / day HA per os16.7, 33.8, 67.5, or 135 mg/kg/day DACT per os 13, 26.7, 53, 106.7, or 213 mg/kg/day propazine per os	VO delay in animals treated with ≥33.8 mg/kg DACT (dose-dependent), or with ≥106.7 mg/kg propazine	25 mg/kg/day for atrazine, 16.7 mg/kg/day for DACT
Zorilla et al., 2010 [66]	Simazine	Female Wistar rats	PND 22–42 or PND 22–62	12.5, 25, 50, 100, or 200 (not administered to the animals treated only for 21 days) mg/kg/day per os.	VO delay for the subgroups exposed at 25 and 100 mg/kg for 21 days, and the subgroups exposed to ≥25 mg/kg for 41 days. Delay of first estrus appearance for the subgroup exposed at 100 mg/kg for 21 days, or at 100 and 200 mg/kg for 41 days.	n/a
Rollerova et al., 2011 [35]	Acetochlor	Female Wistar rats	PND 4–PND 7	7.68 or 15.36 mg/kg/day sc	VO acceleration at all dosages	n/a
Mathias et al., 2012 [36]	Metolachlor	Male Wistar rats	PND 23–PND 53	5 or 50 mg/kg/day per os	Dose-dependent PPS acceleration	23.5–26 mg/kg/day
Romano et al., 2010 [67]	Glyphosate	Male Wistar rats	PND 23–PND 53	5 or 50 or 250 mg/kg per os	Dose-dependent PPS delay at 50 or 250 mg/kg	50 mg/kg/day
			*Gestational and Postnatal*			
Breckenridge et al., 2015 [68]	Atrazine	SD rats	F0 generation: GD 0–LCD 21 F1 generation: PND 21–5 post VO days	6.25, 25, or 50 mg/kg/day per os	VO delay at 25 or 50 mg/kg/day atrazine starting in utero, and at 50 mg/kg/day atrazine starting after weaning	6.25 mg/kg/day
Manservisi et al., 2019 [69]	Glyphosate-based herbicide	Female SD rats	F0 generation: GD 6–end of lactation F1 generation: from weaning and for 13 weeks	175 mg/kg/day per os	Delay of first estrus appearance in F1 generation	50 mg/kg/day
Romano et al., 2012 [38]	Glyphosate	Wistar rats	GD 18–LCD 5	50 mg/kg/day per os	PPS acceleration	50 mg/kg/day
			*Transgenerational*			
McBirney et al., 2017 [37]	Atrazine	Harlan SD rats	F0 generation: GD 8–GD 14	25 mg/kg intraperitoneally	Accelerated puberty onset in F2 generation male and F3 generation female animals	n/a
	**Fungicides**					
			*Postnatal*			
Blystone et al., 2007 [70]	Prochloraz	Male SD rats	PND 23–PND 42; PND 23 –PND 51	31.3, 62.5, or 125 mg/kg/day per os	PPS delay at 125 mg/kg/day	5 mg/kg/day
			*Gestational and Postnatal*			
Schneider et al., 2017 [71]	Mixture of vinclozolin/flutamide/prochloraz	Wistar rats	GD 6–LCD 21 and PND 21–puberty onset; PND 21–83	0.005/0.00025/0.01, 4/0.025/5 or 20/0.25/30 mg/kg/day per os	PPS delay at 20/0.25/30 mg/kg/day	4/0.025/5 mg/kg/day
Melching-Kolmuss et al., 2017 [72]	Prochloraz	Wistar rats	GD 6–LCD 21	0.01, 5, or 30 mg/kg/day per os	PPS delay at 30 mg/kg/day	5 mg/kg/day

NOAEL: no-observed-adverse-effect level; SD: Sprague–Dawley; PND: postnatal day; VO: vaginal opening; sc: subcutaneous; PPS: preputial separation; GD: gestation day; LCD: lactation day; DDT: dichlorodiphenyltrichloroethane; DDE: dichlorodiphenyldichloroethylene; DEA: deethylatrazine; DIA: deisopropylatrazine; DACT: diaminochlorotriazine; HA: hydroxyatrazine.

**Table 3:** Reviewed human studies.

Publications	Agrochemical Substance	Sex, Number (*n*), Country	Age (Years)	Biological Matrice /Method	Impact on Puberty Landmarks
	**Insecticides**				
	*Pyrethroids*				
Ye et al., 2017 [73]	3-PBA (nonspecific metabolite)	Girls (*n* = 305) China	9–15	Urine/LC-MS	Positive association between increased concentration and delay in puberty progression tempo and age at menarche
Ye et al., 2017 [39]	3-PBA (nonspecific metabolite)	Boys (*n* = 463) China	9–16	Urine/LC-MS	Positive association between increased concentration and acceleration in puberty progression tempo
	*Organochlorines*				
Sergeyev et al., 2017 [74]	HCB, βHCH, p,p′-DDE	Boys (*n* = 482) Russia	8–9	Serum/GC-MS	Delayed sexual maturation with HCB
Krstevska-Konstantinova et al., 2001 [45]	p,p′-DDE	Girls and boys (*n* = 41) Multiethnic immigrants (Asians, Africans, South Americans, Western Europeans) and native Belgians	7.8–8.3 (mean age at diagnosis)	Serum/GC-MS/MS	Increased risk for idiopathic precocious puberty among immigrants from developing countries to Belgium
Croes et al., 2015 [40]	HCB, p,p′-DDE	Boys and girls (*n* = 600) Belgium	14–15	Serum/GC-MS	Delayed sexual maturation in girls and accelerated in boys with HCB and delayed sexual maturation in girls with p,p′-DDE
Bapayeva et al., 2016 [75]	Lindane, dieldrin, endrin, DDT	Girls (*n* = 517) Kazakstan	10–17	Serum/GC-ECD	Delayed sexual maturation
Vasiliu et al., 2004 [41]	DDE	Women (*n* = 151) USA	20–50	Maternal serum/GC-ECD	Acceleration of menarche
Ouyang et al., 2005 [42]	DDT	Women (*n* = 466) China	20–36	Serum/GC-ECD	Acceleration of menarche
DenHond et al., 2011 [43]	HCB, p,p′-DDE	Boys (*n* = 767) and girls (*n* = 636) Belgium	14–15	Serum/GC-ECD	Accelerated pubertal development in boys
Grandjean et al., 2012 [76]	p,p′-DDE	Boys (*n* = 438) Faroe Islands	14	Cord blood/GC-ECD	Negative association with pubertal development
Sayied et al., 2003 [77]	Endosulfan	Boys (*n* = 117) India	10–19	Serum/GC-ECD	Delayed pubertal development
Attfield et al., 2019 [78]	DDE HCB Transnonaclor	Girls (*n* = 556) USA (multiracial cohort)	6–8 (age at enrollment)	Serum/GC-MS	Positive association between organochlorine pesticides concentration in the highest quartile and delayed menarche
Deng et al., 2012 [44]	p,p′-DDE	Boys (*n* = 3) and girls (*n* = 175) China	~3–9	Serum/GC-ECD	Positive association between exposure and idiopathic precocious puberty
	*Organophosphates*				
Croes et al., 2015 [40]	DMP, DMTP, DMDTP, DEDTP	Boys and girls (*n* = 600) Belgium	14–15	Urine/GC-MS	Delayed sexual maturation in boys with methyl metabolites and delayed sexual maturation in girls with ethyl metabolites
	**Herbicides**				
Namulanda et al., 2017 [47]	Atrazine metabolites	Girls (*n* = 469) United Kingdom	8–13	Maternal urine during pregnancy (collected at 8th-17th week) /LC-MS/MS)	Positive association between maternal urine DACT concentrations and the risk for earlier menarche among prenatally exposed daughters
	**Pesticide mixture**				
Wohlfahrt-Veje et al., 2012 [46,79]	Various pesticide categories	Boys (*n* = 94) and girls (*n* = 83) and Denmark	6–11	Indirect assessment (questionnaire) of occupational exposure of female greenhouse workers during their first trimester of pregnancy	Earlier thelarche in prenatally exposed daughters and smaller testicular volumes and penile lengths at 3 months of age and prepubertally in prenatally exposed sons

3-PBA: 3-phenoxybenzoic acid; 3-phenoxybenzoic acid, LC: liquid chromatography; MS: mass spectrometry; HCB: hexachlorobenzene; βHCH: beta-hexachlorocyclohexane; DDE: dichlorodiphenyldichloroethylene; GC: gas chromatography; MS/MS: tandem mass spectrometry; DDT: dichlorodiphenyltrichloroethane; ECD: electron capture detector; USA: United States of America; DMP: dimethylphosphate; DMTP: dimethylthiophosphate; DMDTP: dimethyldithiophosphate; DEDTP: diethyldithiophosphate; DACT: diaminochlorotriazine.
